# Genome Sequences of the Race 1 and Race 4 *Xanthomonas campestris* pv. campestris Strains CFBP 1869 and CFBP 5817

**DOI:** 10.1128/genomeA.01023-15

**Published:** 2015-09-17

**Authors:** Stéphanie Bolot, Aude Cerutti, Sébastien Carrère, Matthieu Arlat, Marion Fischer-Le Saux, Perrine Portier, Stéphane Poussier, Marie-Agnes Jacques, Laurent D. Noël

**Affiliations:** a INRA, UMR 441 Laboratoire des Interactions Plantes-Microorganismes (LIPM), Castanet-Tolosan, France; b CNRS, UMR 2594 Laboratoire des Interactions Plantes-Microorganismes (LIPM), Castanet-Tolosan, France; c Université de Toulouse, Université Paul Sabatier, Toulouse, France; d INRA, UMR 1345 Institut de Recherche en Horticulture et Semences (IRHS) SFR 4207 QUASAV, Beaucouzé, France; e Université d’Angers, UMR 1345 Institut de Recherche en Horticulture et Semences (IRHS) SFR 4207 QUASAV, Beaucouzé, France; f Agrocampus Ouest, UMR 1345 Institut de Recherche en Horticulture et Semences (IRHS) SFR 4207 QUASAV, Beaucouzé, France

## Abstract

*Xanthomonas campestris* pv. campestris is the causal agent of black rot on *Brassicaceae*. The draft genome sequences of strains CFBP 1869 and CFBP 5817 have been determined and are the first ones corresponding to race 1 and race 4 strains, which have a predominant agronomic and economic impact on cabbage cultures worldwide.

## GENOME ANNOUNCEMENT

*Xanthomonas campestris* pv. campestris is the causal agent of black rot on a wide range of *Brassicaceae*, including vegetable crops (e.g., cabbages and radishes) or weeds (e.g., *Arabidopsis thaliana*) (1). Nine races of *X. campestris* pv. campestris were defined based on the symptoms caused on a set of host and nonhost plants (2). Although races 1 and 4 are the most prevalent worldwide in infected cabbage fields (1), genomic sequences have only been reported for races 3 and 9 (3, 4).

Strains CFBP 1869 and CFBP 5817 are, respectively, from race 1 and race 4 of *X. campestris* pv. campestris (2). CFBP 1869 was isolated in 1976 in Côte d’Ivoire on *Brassica oleracea* and CFBP 5817 in 2001 in Chile on *Brassica oleracea* var. *Botrytis* (http://www6.inra.fr/cirm_eng/CFBP-Plant-Associated-Bacteria). Strains CFBP 1869 and CFBP 5817 cause typical black rot disease symptoms on cabbages and *Arabidopsis* and belong to genomic clades A and B of *X. campestris* pv. campestris, respectively (5). Shotgun sequencing of total genomic DNA was performed on an HiSeq 2000 Illumina platform. For strains CFBP 1869 and CFBP 5817, 21,031,822 and 58,939,962 paired-end reads of 101 bp were obtained and correspond to 424- and 1,199-fold coverage, respectively. Genome assembly was performed using a combination of SOAP*denovo* (6) and Velvet (7) assemblers and yielded 102 and 109 contigs >500 bp and an *N*_50_ of 142,801 and 124,247 bp, respectively. The average contig sizes are 49,106 and 45,556 bp, the largest being 405,827 and 349,452 bp long, for total genome sizes of 5,008,832 and 4,965,622 bp for strains CFBP 1869 and CFBP 5817, respectively. For each strain, 99 of those contigs were organized in 2 pseudomolecules based on the chromosomal organization of *X. campestris* pv. campestris strain 8004. The largest ones (5,011,687 and 4,918,955 bp; 65% G+C content) correspond to the chromosomes of strains CFBP 1869 and CFBP 5817, respectively. A remaining pseudomolecule of 32 kb for strain CFBP 5817 should account for the ca. 32-kb endogenous plasmid of this strain (5). *De novo* annotation was performed on the remaining areas using Eugene-P (8). Annotation transfer was performed using BLAST best hits and synteny conservation with *X. campestris* pv. campestris strains B100, 8004, and ATCC 33913 as priority references and other publicly available *Xanthomonas* genomes. A total of 4,535 and 4,432 coding sequences (CDSs) were identified for strains CFBP 1869 and CFBP 5817, respectively, as well as 53 tRNA genes and 3 rRNA genes for both.

Sequence analysis of the genomes of strains CFBP 1869 and CFBP 5817 enabled the detection of genes important for pathogenicity, such as the *hrp* gene cluster encoding the type III secretion system (9) and genes encoding the core type III-secreted proteins of *X. campestris* pv. campestris (5). The strains differ in the presence of *xopG*, *xopAC/avrAC*, and *xopAL1* in CFBP 1869 and *xopJ5* in CFBP 5817. The genome sequences of strains CFBP 1869 and CFBP 5817 will improve our knowledge of intraspecific genomic diversity in *X. campestris* pv. campestris and should allow a more rational breeding of resistance to black rot disease in cultivated *Brassicaceae*.

### Nucleotide sequence accession numbers.

The genome assemblies for CFBP 1869 and CFBP 5817 have been deposited at NCBI under the accession numbers AVDD00000000 and AVDL00000000, respectively.
